# The Circadian System Is a Target and Modulator of Prenatal Cocaine Effects

**DOI:** 10.1371/journal.pone.0000587

**Published:** 2007-07-11

**Authors:** Eva H. Shang, Irina V. Zhdanova

**Affiliations:** Department of Anatomy and Neurobiology, Boston University School of Medicine, Boston, Massachusetts, United States of America; University of Massachusetts, United States of America

## Abstract

**Background:**

Prenatal exposure to cocaine can be deleterious to embryonic brain development, but the results in humans remain controversial, the mechanisms involved are not well understood and effective therapies are yet to be designed. We hypothesize that some of the prenatal effects of cocaine might be related to dysregulation of physiological rhythms due to alterations in the integrating circadian clock function.

**Methodology and Principle Findings:**

Here we introduce a new high-throughput genetically well-characterized diurnal vertebrate model for studying the mechanisms of prenatal cocaine effects by demonstrating reduced viability and alterations in the pattern of neuronal development following repeated cocaine exposure in zebrafish embryos. This effect is associated with acute cocaine-induced changes in the expression of genes affecting growth (growth hormone, *zGH*) and neurotransmission (dopamine transporter, *zDAT*). Analysis of circadian gene expression, using quantitative real-time RT-PCR (QPCR), demonstrates that cocaine acutely and dose-dependently changes the expression of the circadian genes (*zPer-3, zBmal-1*) and genes encoding melatonin receptors (*zMelR*) that mediate the circadian message to the entire organism. Moreover, the effects of prenatal cocaine depend on the time of treatment, being more robust during the day, independent of whether the embryos are raised under the light-dark cycle or in constant light. The latter suggests involvement of the inherited circadian factors. The principal circadian hormone, melatonin, counteracts the effects of cocaine on neuronal development and gene expression, acting via specific melatonin receptors.

**Conclusions/Significance:**

These findings demonstrate that, in a diurnal vertebrate, prenatal cocaine can acutely dysregulate the expression of circadian genes and those affecting melatonin signaling, growth and neurotransmission, while repeated cocaine exposure can alter neuronal development. Daily variation in these effects of cocaine and their attenuation by melatonin suggest a potential prophylactic or therapeutic role for circadian factors in prenatal cocaine exposure.

## Introduction

Tens of thousands of babies that have been exposed to cocaine in utero are born in the United States every year [1]. Multiple human studies suggest significant changes in brain development and subsequent brain function in children of drug-addicted parents [2], [3]. However, the extent of the damage and whether it is in part due to confounding environmental, genetic or psychological factors remains controversial [4]. In contrast, studies in animal models, including rodents and primates, clearly show that prenatal cocaine exposure has deleterious effects on the developing brain, affecting both cognitive functions and emotional responses [5], [6].

We hypothesize that inter-individual variability in the prenatal effects of cocaine might reflect an irregular pattern of exposure of human embryos to cocaine, self-administered by their mothers at different times of day, at irregular intervals and at different doses. Such intermittent cocaine use could produce variable results in affecting the circadian clock, known to respond differently to drugs or environmental stimuli depending on the time of exposure [for review, 7]. Alterations in the integrating role of the clock system would result in mutual de-synchronization of internal physiological processes and lack of coordination with the external, including maternal, environment. Considering the role of the circadian system in the control of cell division [8]–[10], especially important during early development, altered clock mechanisms might lead to multiple subtle embryonic abnormalities. While studies in rodents suggest that the circadian system has close links to the behavioral effects of cocaine in adults [for review, 11], no research on whether cocaine affects the circadian-related processes during early development has yet been done.

To address our hypothesis, we chose a popular animal model of vertebrate development and genetics, the zebrafish. Translucent zebrafish embryos allow for direct visual monitoring of a live and developing vertebrate. This and genetically encoded fluorescent reporters (e.g., green fluorescent protein, GFP) in transgenic zebrafish provide a unique opportunity for studying expression of specific genes throughout development. To assess potential differences in zebrafish brain development in response to cocaine, we have used α1-T-GFP transgenic zebrafish, with a GFP reporter driven by the 1.696-kb goldfish α1-tubulin promoter fragment [12]. α1-Tubulin is a neural-specific isoform of the α-tubulin family of proteins, forming the microtubule cytoskeleton in developing axons and dendrites [for review 13]. The reporter recapitulates α1-tubulin endogenous gene expression and thus is present in committed neuroprogenitors and actively developing neurons, allowing qualitative and quantitative evaluation of the developing brain and spinal cord [12]–[14].

An additional advantage of our animal model for understanding the role of the circadian system in human physiology is that zebrafish are diurnal vertebrates, and exhibit a temporal relationship between circadian factors and the physiological states similar to that in humans. In spite of phylogenetic conservation of the circadian genes and overall mechanisms of the circadian clock, the functional relationship between the day/night circadian phases and the physiological or behavioral status of the organism differs between diurnal and nocturnal species [for review, 15]. For example, the neuronal activity of the mammalian master clock, the suprachiasmatic nucleus of the hypothalamus (SCN), is high during the day in both diurnal and nocturnal species. However, an exclusively nighttime secretion of the principal hormone of the circadian system, melatonin, coincides with active behavioral and physiological phase in nocturnal species, while diurnal species, including humans, are at rest or sleep when their melatonin is at peak. Accordingly, the circadian factors, including melatonin can have distinctly different effects in diurnal and nocturnal animals, e.g., melatonin can promote sleep in only diurnal vertebrates [for review, 16].

Another important advantage of zebrafish for circadian-related studies on development is that their clock mechanisms are active early in embryogenesis, with variation in some circadian gene expression (e.g., *zPer-3*) being under control of inherited factors [17] and others requiring development of the embryonic circadian clock [for review, 18]. In zebrafish, the principal circadian hormone, melatonin, and its receptors are functional by the end of the first day post-fertilization [10]. The six zebrafish melatonin receptors identified are homologous to those found in mammals, amphibians or birds [19]: *zMel1a-1, zMel1a-2* and *zMel1a-3* (corresponding to *Mel1a* or *MT1* in other species), *zMel1b-1* and *zMel1b-2* (corresponding to *Mel1b* or *MT2*), and *zMel1c (Mel1c)*.

Early embryonic expression of functional melatonin receptors and their much higher levels in the embryos of several species studied, including humans, suggest that melatonin and the overall circadian system play an important role in development [for review, 20]. Our earlier finding that melatonin promotes zebrafish embryogenesis and cell division [10] further supports this notion. Importantly, in humans, maternal melatonin acting through its specific receptors might be the principal circadian factor available to an embryo, suggesting that alterations in melatonin levels or melatonin receptor expression might significantly affect human development.

Here we report that prenatal exposure to cocaine, in concentrations comparable to those experienced by human embryos, alters neuronal development in zebrafish and is associated with acute changes in the embryonic expression of mRNA for growth hormone and the dopamine transporter. These effects of prenatal cocaine exhibit significant circadian variation and involve changes in the expression of the inherited and embryonic clock genes, as well as the genes encoding melatonin receptors. Moreover, melatonin pre-treatment attenuates or blocks the effects of cocaine on neuronal development and embryonic gene expression. Thus, the circadian system might be at the core of the developmental effects of cocaine and their inter-individual variability. Circadian factors, including melatonin, could provide new therapeutic strategies to counteract the developmental effects of prenatal cocaine exposure.

## Results

### Daytime cocaine exposure affects embryonic mortality rate, neuronal development and expression of genes responsible for growth and neurotransmission

The choice of cocaine concentrations used in our experiments was based on those experienced by the human fetuses of cocaine-addicted mothers. The 0.3 µM cocaine concentration (referred to as Low-dose Cocaine below) is similar to human plasma cocaine levels following injection of a low 0.2 mg/kg (i.v.) cocaine dose capable of changing mood and physiological parameters [21]. Similar maximal umbilical cord plasma cocaine levels were documented in neonates [22], presumably hours after their mothers self-administered cocaine.

To confirm that cocaine can be absorbed by the zebrafish during development, cocaine levels were measured at 6 days post fertilization (dpf). Following a single 15-min immersion in cocaine solution, cocaine levels in larvae corresponded to 0.4–0.6 ng per 100 µg protein and 9–12 ng per 100 µg protein for the 1.5 and 30 µM doses, respectively. Measuring tissue cocaine levels in de-chorionated zebrafish embryos was impractical due to the very large number of subjects per sample required to achieve the assay sensitivity threshold. The degree of cocaine absorption can be expected to be the same or higher in the de-chorionated embryos, compared to larvae. Based on these results, the study was conducted in de-chorionated embryos (or larvae), mainly using the 0.03–30 µM range of cocaine doses administered via immersion.

The embryonic mortality rate was not affected following a single 15-min cocaine treatment, however it was dose-dependently increased in de-chorionated zebrafish embryos repeatedly treated with cocaine during the daytime (starting ZT 5, 22 hpf, 15 min per hour for 5 hours). The higher doses of cocaine resulted in higher mortality rate within 24-h after treatment. While in the control de-chorionated embryos the mortality rate was 4–9 per 100 embryos, cocaine treatment increased this rate 6±2.1% and 37±6.3% above control level following 1.5 µM and 30 µM dose, respectively (p<0.05 for both, based on paired t-test of within clutch comparisons, six individual clutches per treatment). An intact chorion protected the zebrafish embryos from cocaine (no difference from control), although cocaine could induce earlier hatching of the embryos. The latter suggests increased cocaine penetration during pre-hatching changes in the chorion.

**Figure 1:**
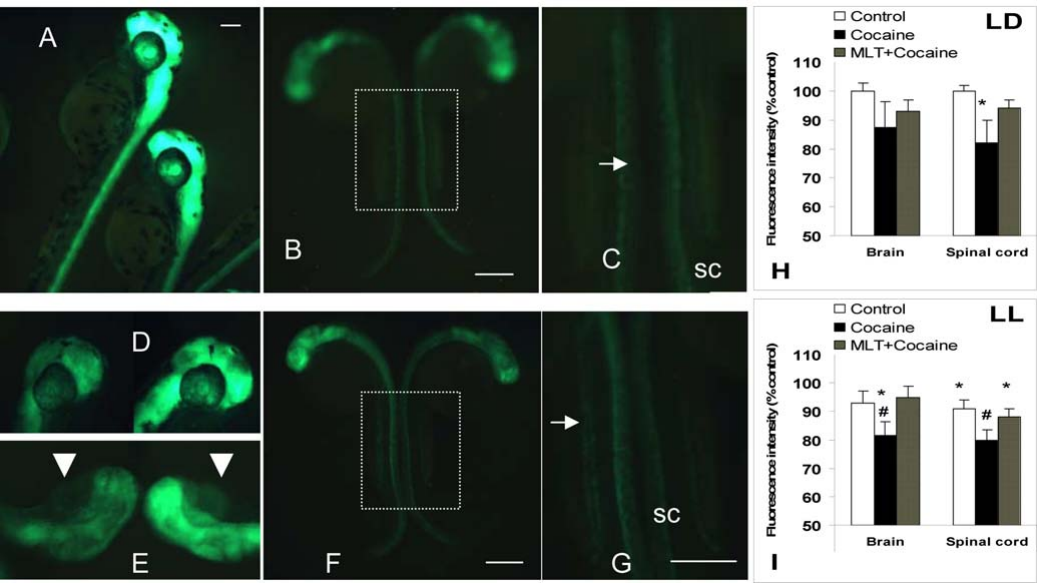
Prenatal exposure to cocaine alters neuronal development in α1-TGFP transgenic zebrafish embryos. Control embryos (A: 63 hpf; B and C: 37 hpf) show highly similar patterns of GFP reporter expression. Although major anatomical abnormalities were not found in cocaine-treated embryos, they display variable patterns and typically lower levels of GFP reporter expression (D–G) following repeated cocaine administration (1 µM, 15 min every hour for 5 daytime hours, starting at 22–24 hpf). (C) and (G) correspond to the dotted line areas in (B) and (F), respectively, and show GFP reporter expression in the spinal cord (sc) and enteric neurons (arrows). Note that a double string of enteric neurons, separated by a prospective lumen, is visible in both control embryos (c) but only in one of the cocaine-treated embryos (left in F and G). (D) and (E) depict different GFP expression patterns in the forebrain of four cocaine-treated embryos (37 hpf): lateral (D) and semi-dorsal view (e; arrowheads-eyes). All pictures are original photographs of live, responsive-to-touch embryos of the same clutch, placed next to each other on the cover glass. Black spots in (A) and (D) represent pigment. Out-of-focus areas on the close-up whole-embryo pictures (e.g., B, F) are due to the curved position of the embryo around the spherical yolk sack. The pictures are from one of the 12 clutches studied in LD, and are representative of 3 independent experiments. Scale bars: 250 µm. (H and I) Mean (SEM) percent change in fluorescence intensity in the brain and spinal cord of 37 hpf embryos raised in LD (H) or LL (I) conditions, relative to mean Control group intensity in LD (100%). Control (white bar), repeated cocaine (1 µM) administration alone (black bar) or with 30-min melatonin pre-treatment (diagonal bar) at 22 hpf. p<0.05 * relative to LD Control, or # relative to LL Control (one-way ANOVA), n = 28 per treatment group.

Upon inspection, the surviving cocaine-treated embryos or larvae typically showed no apparent signs of anatomical abnormalities following repeated cocaine exposures (0.3, 1 or 3 µM), compared to control. Consistent with the original observations [12], [14], α1-tubulin-associated GFP fluorescence in control α1-TGFP zebrafish embryos was first detected 12–14 hours post fertilization (hpf) and, within a few hours, highlighted the entire central nervous system (CNS), thereafter peaking in different regions in a predictable pattern. The control group showed highly similar levels of fluorescence (Fig. 1 A–C). In contrast, cocaine-treated embryos demonstrated multiple changes in GFP fluorescence, ranging from an overall reduction or, less frequently, an overall increase in neuron-associated fluorescence. The areas affected included the forebrain (Fig. 1 A vs. D, E), spinal cord and enteric neurons (Fig. 1 B, C vs. F, G), with the degree varying between and within the individual clutches of embryos. Moreover, some of the embryos that were viable and responsive to touch showed localized changes, with GFP signal being increased or decreased only in some areas of the CNS, compared to control. This suggested that the pattern of brain development has been changed by cocaine exposure.

By the end of the first day post-fertilization, zebrafish embryos are actively developing neuronal structures, analogous to those targeted by cocaine in mammals, forming the dopaminergic, serotoninergic and noradrenergic neuronal systems [23], [24]. At the same time, the circadian structures, such as the pineal gland and retina, undergo fast growth and differentiation [25]. To address whether cocaine-induced changes in neuronal patterns observed in α1-TGFP embryos could involve a broader phenomenon of growth-related processes or alterations in neurotransmission, changes in the mRNA levels for growth hormone (*zGH*) and the dopamine transporter (*zDAT*) were quantified. The latter gene was especially interesting because its protein plays a principal role in cocaine effects in adults and potentially contributes to the effects of prenatal cocaine on reward responses during adulthood.

**Figure 2:**
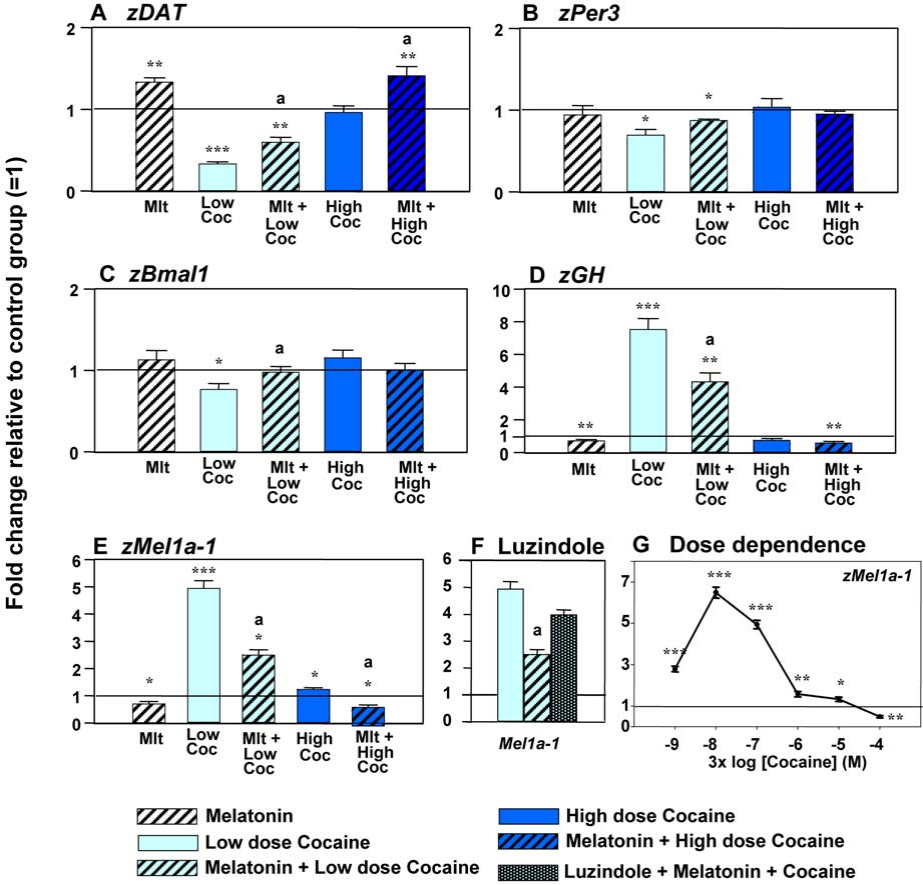
Prenatal daytime cocaine exposure dose-dependently alters expression of genes involved in neurotransmission (A) circadian system (B, C) growth (D) and melatonin signaling (E), and these effects are counteracted by melatonin. (A–E) Melatonin (100 nM for 45 min), Low (0.3 µM) or High (30 µM) cocaine doses (15 min), with or without melatonin pre-treatment (100 nM, 30 min). (F) Melatonin receptor antagonist Luzindole attenuates the counteracting effects of melatonin on cocaine-induced changes in the expression of all six melatonin receptor genes; only one (*zMel1a-1*) receptor shown. Treatments: luzindole 50 µM for 20 min prior to melatonin; melatonin 100 nM for 20 min prior to cocaine; cocaine 0.3 µM for 15 min. (G) Dose-dependent effects of cocaine (20 min) on melatonin receptor expression. Y axis: fold change relative to control ( = 1). N = 3–4 samples/treatment group, 25 embryos/sample. Mean±SEM, *t*-test (vs. control), * *p*<0.05, ** *p*<0.01, *** *p*<0.001; One way ANOVA (between treatments), a: *p*<0.05, vs. cocaine-only group. Mlt-melatonin; Coc-cocaine.

A single 15-min exposure to 0.3 µM cocaine at 24 hours post-fertilization (hpf) significantly (p<0.001) altered the daytime expression of *zGH*, causing a 7.5-fold upregulation (Fig. 2D). At the same time, *zDAT* expression was inhibited (p<0.001) by this dose of cocaine (Fig. 2A). In contrast, similar exposure of the zebrafish embryos to a 100 times higher dose of cocaine (30 µM), that increases their mortality rate, failed to produce significant changes in the expression of both genes. It should be noted that these and other doses used in this study did not affect the expression of the control gene (β-actin) or total mRNA levels.

### Cocaine alters the daytime expression of mRNA for circadian genes and melatonin receptors

A single 15-min exposure to cocaine significantly and dose-dependently altered the daytime expression of the mRNA for the circadian genes (*zBmal-1, zPer-3*) and six melatonin receptors (*zMelR*) (Fig. 2). The low dose (0.3 µM) of cocaine reduced *zBmal-1* and *zPer-3* expression, while causing an up to 4-fold increase in mRNAs for six *zMelR* genes, including *Mel1a-1* (Fig. 2 B, C, E). Similar to what was found for *zGH* and *zDAT*, increasing the dosage reduced cocaine efficacy in changing the gene expression, independent of the initial direction of the effect observed. For example, the low 0.3 µM dose inhibited *zPer-3* or *zBmal-1* expression, with no effect of the 30 µM treatment on both genes (Fig. 2 B–C). Similarly, the dose increase caused gradual attenuation of cocaine-induced over-expression of all six *zMelR* genes, and even reversed the response, to slight inhibition, following exposure to the maximal, 300 µM, dose tested (Fig. 2 E, G). This effect of the higher doses was not a result of a transitory upregulation followed by compensatory downregulation of expression. The higher cocaine doses caused stronger inhibition of gene expression within 5 min of administration, followed by gradual recovery to the control levels (data not shown).

### Daily variation in cocaine effects during development

We then compared the results of daytime and nighttime prenatal exposure to cocaine. Examination of the level and pattern of GFP signals in the neuronal structures of the α1-TGFP zebrafish following repeated nighttime cocaine treatment (0.3 or 30 µM, at 32–37 hpf) showed an improved survival rate and less variability in GFP signal (p<0.01 and 0.0001, respectively; data not shown). However, we had to consider that embryos were 12-h older at the start of the nighttime treatment and the GFP signal by this time had reached a high level in all the embryos. Considering that the half-life of GFP is close to 24-h, these results could be significantly affected by GFP synthesis prior to treatment and might not necessarily reflect daily variation in the effects of cocaine. These results are thus only suggestive of daily variation in the effects of cocaine on neuronal development.

**Figure 3:**
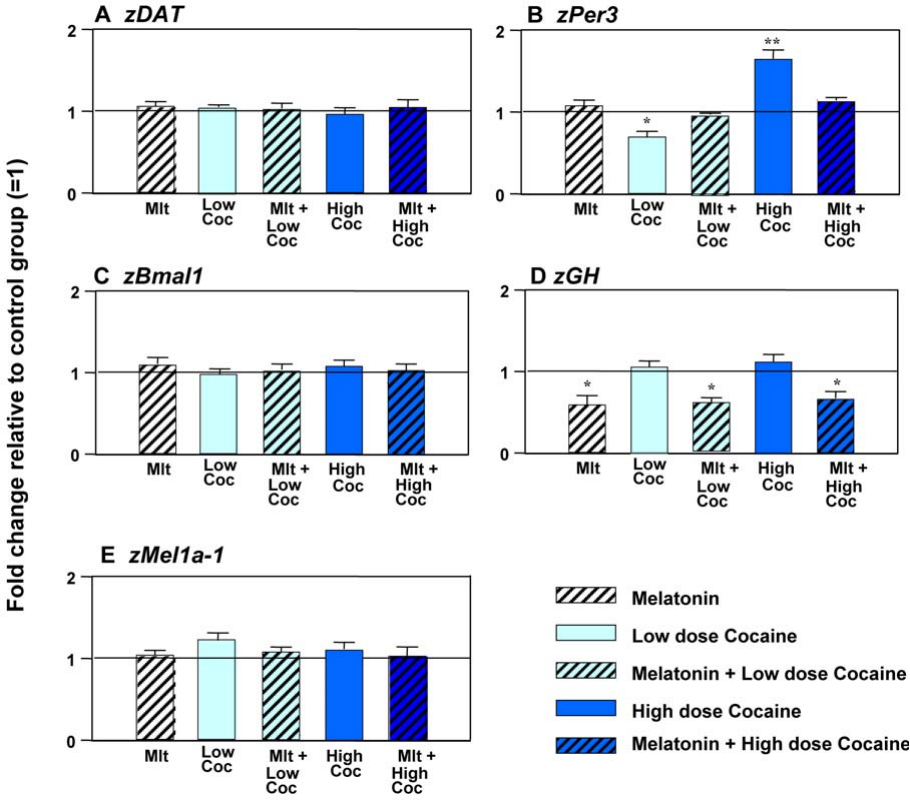
Nighttime low- or high-dose cocaine exposure fails to alter the expression of *zDAT, zBmal1, zGH* and melatonin receptor genes during zebrafish development but continues to affect *zPer3*, and this effect is counteracted by melatonin. (A–E) Melatonin (100 nM for 45 min), Low (0.3 µM) or High (30 µM) cocaine doses (15 min), with or without melatonin pre-treatment (100 nM, 30 min) administered to 36-hpf embryos. N = 3–4 samples/treatment group, 25 embryos per sample. Mean±SEM, *t*-test (vs. control), * *p*<0.05, ** *p*<0.01, *** *p*<0.001; One way ANOVA (between treatments), a: *p*<0.05, vs. cocaine-only group. Mlt-melatonin; Coc-cocaine.

The effects of acute cocaine exposure on gene expression also showed circadian variation. In contrast to the daytime effects of cocaine described above, nighttime cocaine treatment (at 36 hpf, ZT 19) in embryos raised in a light-dark cycle (LD) failed to change the expression of the majority of the genes tested (Fig. 3). The only exception was *zPer-3* expression. While neither low nor high doses of cocaine affected the daytime *zPer-3* mRNA level (Fig. 2 B), the 0.3 µM dose inhibited and 30 µM dose upregulated it at night (Fig. 3 B).

**Figure 4:**
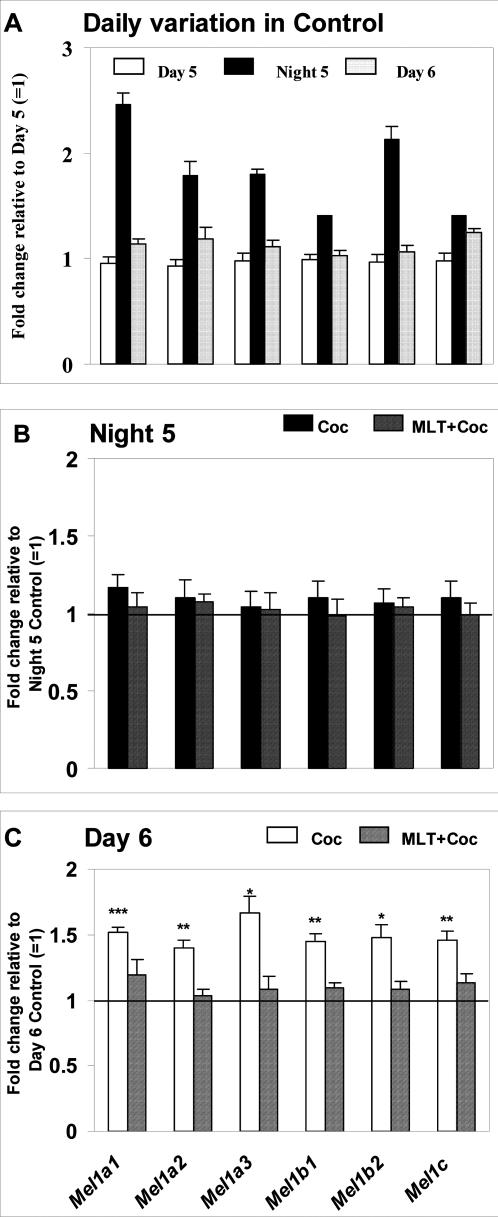
Daily variation in melatonin receptor expression, cocaine efficacy and ability of melatonin to counteract the effects of cocaine are preserved during early postnatal period in zebrafish. (A) Normal daily pattern of mRNA expression for six melatonin receptors on Day 5, Night 5 and Day 6 post-fertilization in Control group, fold change expressed relative to Day 5 ( = 1). (B) Change in melatonin receptor expression following Daytime cocaine administration alone (Coc) or following melatonin pre-treatment (MLT+Coc) at 6 dpf, relative to Daytime Control group ( = 1); (C) Change in melatonin receptor expression following Nighttime cocaine administration alone (Coc) or following melatonin pre-treatment (MLT+Coc) at 6 dpf, relative to Nighttime Control group ( = 1). Cocaine (0.3 µM, 20 min), melatonin pre-treatment (100 nM, 30 min prior to cocaine). Y axis: fold change relative to corresponding control ( = 1). N = 3–4 samples/treatment group, 20 larvae per sample. Mean±SEM, *t*-test (vs. control), * *p*<0.05, ** *p*<0.01, *** *p*<0.001 vs. cocaine-only group; One way ANOVA.

Although the focus of this study was on the prenatal effects of cocaine, we were concerned that fast embryonic development may affect our daytime versus nighttime comparisons. To test this, we assessed the effects of the daytime and nighttime cocaine treatments (ZT 7 or 19) in 5-6-day old zebrafish larvae, at the time when developmental processes slow down, compared to the embryonic stage. Similar to the results obtained in embryos, larvae displayed a circadian pattern of gene expression and cocaine effects, the latter being limited to daytime, as illustrated in Figure 4 for six *zMelR* genes. Combined, these data suggested that nighttime circadian factors are able to neutralize some effects of cocaine during early zebrafish development.

### Melatonin counteracts the embryonic effects of cocaine at daytime and at night

One of the hallmarks of the nocturnal physiological environment is the presence of the principal circadian hormone melatonin, secreted into the blood and cerebro-spinal fluid by the pineal gland. In zebrafish, the pineal axonal projections are among the first to develop, with melatonin production and expression of melatonin receptors being initiated around 18 hpf, and soon after modulating the rate of zebrafish embryonic development and cell division [10]. When the embryos were pre-treated with melatonin 30 min before each of the five consecutive daytime cocaine administrations, the survival rate was improved, showing no difference from control following 1.5 µM cocaine dose, although remained significantly higher than in control after 30 µM cocaine treatment (29±2.7% above control; p<0.05).

Melatonin administered alone produced modest, if any, changes in daytime expression of the genes studied (Fig. 2 A–E, first column). However, when used as a 30-min pre-treatment, melatonin significantly modified the acute daytime effects of cocaine, bringing the expression levels closer to the control or melatonin-only group (Fig. 2 A–E, column 3 and 5). Such effects of daytime melatonin pre-treatment on gene expression were abolished by the specific melatonin receptor antagonist, luzindole, illustrated for *zMel1a-1* (Fig. 2 F). This indicated that specific melatonin receptors mediate this effect of melatonin.

Nighttime administration of melatonin to the embryos raised in LD, i.e., at the time of their normal melatonin production, did not significantly affect any of the genes studied, except for a modest but significant decline in *zGH* expression (Fig. 3 D). When melatonin was used as a pre-treatment, it did not affect gene expression if it was not altered by nighttime cocaine, as in case of the *zDAT, zBmal-1* or six *zMelR* (Fig. 3 A, C, E). Both the downregulation of *zPer-3* by a low (0.3 µM) cocaine dose and its upregulation by a high (30 µM) cocaine dose at night were blocked by melatonin (Fig. 3 B). Similar effects of melatonin and their daily variation were observed in 6-day old larvae (Fig. 4 B,C). Thus, melatonin is at least one of the nocturnal circadian factors that can attenuate or block the effects of nighttime cocaine administration during zebrafish development.

### Circadian variation in prenatal effects of cocaine and melatonin is preserved in the absence of light-dark cycle and embryonic circadian system development

It has been well established that zebrafish do not develop embryonic circadian system while raised in either constant darkness (DD) or constant light (LL), although both conditions eliminate an entrainment to light-dark cycle and preserve the inherited circadian factors [26]–[29]. Thus, the LL conditions allowed us to prevent embryonic entrainment to the environmental time cues, while permitted visual inspection of the embryos, removal of their chorion prior to treatment, and other needed procedures to ensure high quality of the experimental sample collected.

Importantly, while melatonin production can be preserved in DD-raised zebrafish embryos, though lacks circadian variation, LL conditions suppress melatonin secretion [for review, 18]. Thus, LL allowed us to test the effects of cocaine in the absence of both the embryonic circadian system and endogenous melatonin. However, we had to consider that zebrafish embryos inherit some of their circadian factors, as has been shown for *zPer-3* [17], and that LL may alter some circadian parameters. Quantification of mRNA levels for rhythmically expressed circadian genes, *zPer-3* and *zBmal-1*, and six *zMelR,* helped to characterize the impact of environmental illumination on the circadian system in the embryos tested. The daytime and nighttime treatment in LL was administered according to the parents' LD schedule and at the same age as in the control embryos raised in LD.

The mortality rate in control and cocaine-treated embryos tended to be increased when the embryos were maintained in LL, relative to LD, but the change did not reach the level of significance (p = 0.069). The GFP fluorescence intensity was lower in LL-raised α1-TGFP zebrafish embryos. Changes in fluorescence signal were observed in both control (spinal cord areas only) and cocaine-treated zebrafish, compared to LD (Fig. 1 H vs. I). These relatively small changes, however, might be, at least in part, due to the photobleaching effect of continuous bright light exposure.

**Figure 5:**
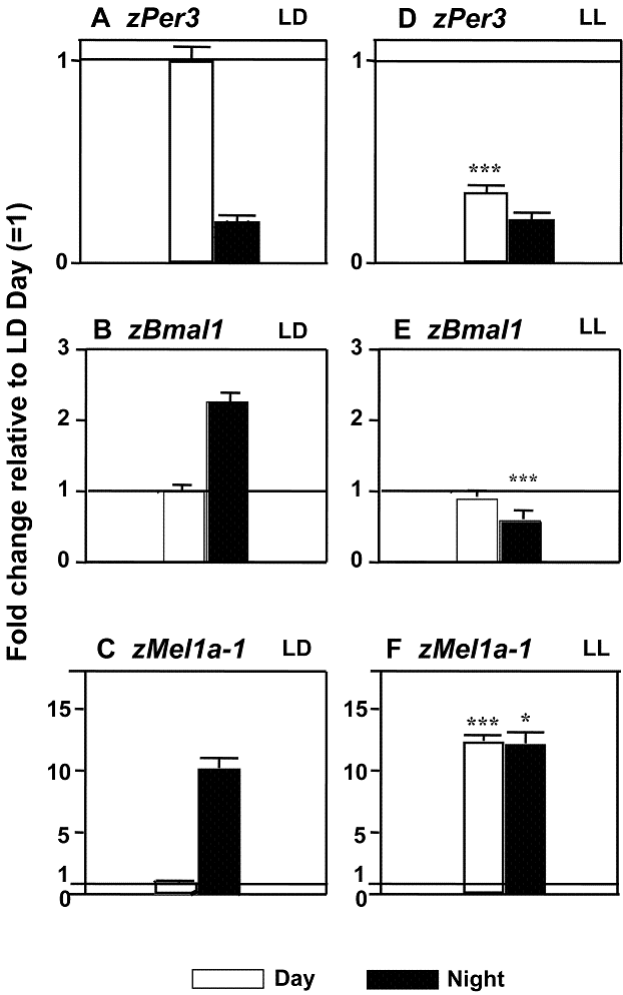
Normal diurnal variation in *zPer3, zBmal1* and melatonin receptors is altered in embryos raised under constant light conditions. Daytime increase in *zPer3* expression in LD (A) is partially preserved in LL (D; *p*<0.05). Daily variation in *zBmal1* and *zMel1a-1* (and other melatonin receptors) is abolished in LL (B vs. E; C vs. F). Y-axis in all plots: fold change relative to LD-Day level ( = 1). N = 3–4 samples/time point in LD or LL group, 25 embryos/sample. Mean±SEM, *t*-test, * *p*<0.05, ** *p*<0.01, *** *p*<0.001 indicates difference between LD and LL, at Day or Night, accordingly.

**Figure 6:**
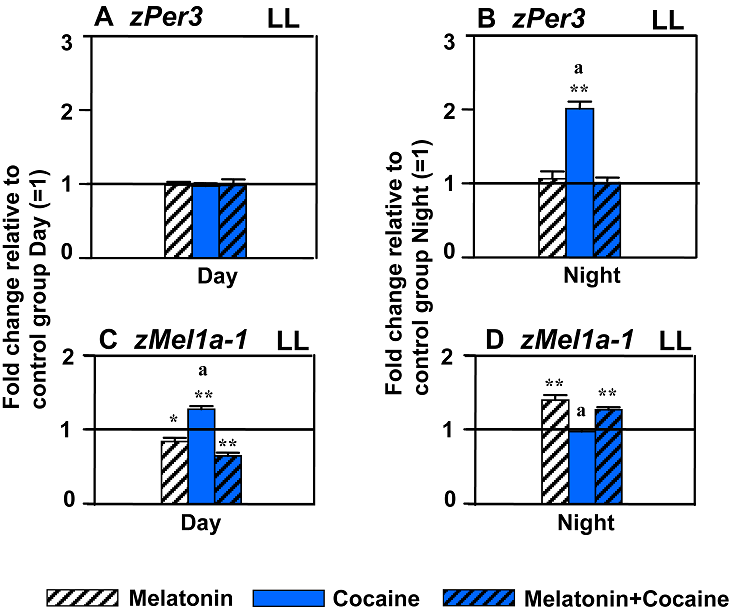
Constant light (LL) changes embryonic gene expression and magnitude of cocaine effects, while preserving circadian variation in cocaine and melatonin actions. Effects of melatonin, cocaine or their combination on gene expression display diurnal (circadian) variation in LL, similar to that in LD (Fig. 2), for both *zPer-3* (A vs. B) or melatonin receptors (C vs. D). Treatment: Melatonin, 100 nM, 50 min or 20 min before cocaine. Cocaine: 30 µM, 20 min. Y-axis: fold change relative to corresponding, Day or Night, LL control ( = 1). N = 3–4 samples/time point in LD or LL group, 25 embryos/sample. Mean±SEM, *t*-test (vs. control), * *p*<0.05, ** *p*<0.01, *** *p*<0.001; One way ANOVA (between treatments), a: *p*<0.05, vs. other two groups.

In control embryos raised in LL, the absolute levels and circadian patterns of gene expression were altered, relative to LD (Fig. 5). The diurnal variation in expression remained present, though strongly reduced, for *zPer-3* (Fig. 5 A, D), while it disappeared for *zBmal-1* (Fig. 5 B, E) and *zMelR* genes, including *zMel1a-1* (Fig. 5 C, F). This pattern of changes confirmed earlier observations of LL blocking the development of the embryonic circadian system but preserving an inherited *zPer-3* variation in expression [17]. In spite of such major changes in the circadian clock in LL, the effects of cocaine continued to display circadian variation, with only nighttime changes in *zPer-3* expression (Fig. 6 A, B) and only daytime changes in *zBmal-1* (data not shown) or *zMelR* (Fig. 6 C, D) mRNA levels.

In LL, in the absence of endogenous melatonin production [18], melatonin treatment augmented nighttime expression of melatonin receptors (Fig. 6D), suggesting that normally, in LD, endogenous melatonin is part of the mechanism promoting circadian variation in melatonin receptor expression. Otherwise, the effects of melatonin in LL, whether administered alone or as a pre-treatment, were similar to those in LD for both nighttime and daytime administration. Accordingly, melatonin continued to slightly downregulate *zMelR* in the daytime (Fig. 6C) and blocked cocaine-induced upregulation of *zPer-3* expression at night and *zMelR* during the daytime (Fig. 6 B, C). Thus, daily variation in the effects of cocaine and melatonin during early development was preserved in the absence of the embryonic circadian system or nighttime melatonin secretion. Considering that no environmental circadian cues were available to the embryos raised in LL, with temperature and other parameters being constant, it appears that inherited circadian factors might be sufficient to modulate the effects of cocaine and melatonin in zebrafish embryos.

## Discussion

Understanding the mechanisms involved in prenatal cocaine effects and finding the means to counteract them is important for both medical research and clinical practice. In this study we present evidence that cocaine alters the expression of at least two principal circadian genes of the *Bmal* and *Per* families, and genes encoding for melatonin receptors, that mediate the circadian message throughout the organism. This supports our original hypothesis that prenatal effects of cocaine and their inter-individual variability may involve the integrating circadian system, potentially causing desynchronization of multiple developmental events. It remains to be seen whether these effects of cocaine alter the intrinsic clock itself or only affect the downstream circadian-controlled processes.

The highly diverse neuronal phenotype of α1-T-GFP embryos repeatedly treated with cocaine suggests that zebrafish neuronal development can suffer from cocaine exposure. While this effect varies depending on the time of treatment and melatonin pre-treatment could to some extent attenuate it, a potential link between the circadian effects of cocaine and neurodevelopment remains to be fully elucidated. Considering that zebrafish, the diurnal vertebrates with well-characterized circadian genetics and circadian mutants available [30], demonstrate both effects, they can provide a useful model for addressing our hypothesis and studying an overall impact of prenatal cocaine.

Need for repeated prenatal cocaine administration to induce changes in neuronal pattern may reflect an accumulated toxic effect or, if our hypothesis is correct, a more pronounced desynchronization of intrinsic processes following repeated assault. Clarifying this issue and studying microscopic changes associated with the observed alterations in the expression of the α1-tubulin-associated reporter will be a subject of further investigation. Considering that neuron-specific α1-tubulin transcription spans the entire period of neuron development, from the neuroprogenitor stage to the completion of axonogenesis [12], [31], [32], alteration in all or some of these stages could result in changed intensity or patterns of fluorescence.

While changes in neuronal pattern require repeated cocaine administration, even a single brief exposure to cocaine dramatically alters the expression of growth hormone mRNA, increasing it up to 800% in zebrafish embryos. At the same time, the expression of the dopamine transporter gene is reduced, following brief exposure to cocaine, to around 40% of that in the control embryos. Considering the important and diverse roles of these two proteins, repeated cocaine-induced changes in their expression levels are likely to result in multifactorial effects on neuronal development. We speculate that these changes might have contributed to the neuronal pattern alterations in cocaine-treated α1-T-GFP embryos.

The results of this study for the first time demonstrate that the circadian system, embryonic and/or inherited, is not only a target of prenatal cocaine exposure but also a modulator of prenatal cocaine effects, with the impact of cocaine on embryonic and larval gene expression depending on the time of day. This is consistent with earlier observations on the day-night differences in the behavioral effects of cocaine in adult invertebrates [33] and vertebrates [34], [35], either wild type or circadian gene mutants, suggesting a close link between cocaine's effects and the circadian clock [11].

Because poor penetration of cocaine through zebrafish chorion requires removing a chorion under the light microscope, constant dark conditions (DD) were not used in this study. The LL conditions, similar to DD, prevent development of embryonic circadian rhythms but preserve inherited circadian factors in the absence of environmental time cues [17]. Daily variation in cocaine effects in LL and their attenuation by the nocturnal circadian hormone, melatonin, strongly suggest that the intrinsic circadian factors can be sufficient to modulate the prenatal effects of cocaine. However, the results of the experiments conducted in LL, when bright light suppresses melatonin production, also demonstrate that melatonin is only one of the intrinsic circadian factors that can modulate the effects of cocaine. Other factors, those that maintain their rhythmic function in LL and presumably inherited by the embryo, would be the primary candidates for such effects.

The nighttime reduction in cocaine efficacy and the ability of melatonin to counteract the effects of cocaine on embryonic gene expression or embryonic survival, also suggests that circadian factors could potentially serve as preventive or therapeutic agents. Melatonin has an impressively low toxicity, based on the lack of side effects in animal and human studies using low or high doses of the hormone [for review, 16]. However, its effects on human development are virtually unknown. Further in-depth studies in diurnal species are required to evaluate all the aspects of the potential effects of melatonin on embryonic development and cautious approach to using melatonin or melatonin receptor agonists during pregnancy is advised.

A biphasic dose-dependence of cocaine effects, similar to those observed in our study, is an interesting phenomenon repeatedly documented in diverse experimental paradigms, although the mechanisms involved remain to be fully understood. For example, hippocampal long-term potentiation (LTP) in rats is not affected by 1 or 3 µM cocaine, increased by 5 and 10 µM, with the effect declining at 20 µM and changing to inhibition at 30 µM [36]. The results of the latter study suggested that the enhancing effects of cocaine on LTP are primarily due to blockade of monoamine transporters, while an inhibitory effect of high cocaine doses is related to its blockade of sodium channels, similar to its local anesthetic mechanism. Increased mortality rate and lack of major changes in gene expression following high dose cocaine treatment in our zebrafish embryos may suggest a non-specific, toxic or deep anesthetic effect of cocaine, blunting the physiological responses to the drug.

On the other hand, the cocaine concentrations that, according to our study, induce maximal changes in gene expression or alter neuronal patterns correspond to those that are experienced by the human embryos of cocaine-addicted mothers [21], [22]. This further suggests that the powerful arsenal of methodological approaches, and the transgenic and mutant phenotypes available for zebrafish research, provides a promising avenue for the study of prenatal effects of cocaine in humans. The high throughput capabilities of this model should help in designing new therapeutic approaches to prevent cocaine-induced developmental alterations.

## Materials and Methods

### Zebrafish maintenance and embryo collection

Wild-type zebrafish (*Danio rerio*, wild-type AB strain) and α1-TGFP transgenic zebrafish [12], were used in this study. The α1-TGFP transgenic zebrafish carries a GFP reporter driven by the α1-tubulin promoter that is expressed in actively developing neuronal structures [12], [14] fish, we developed an α1-TGFP transgenic fish in our lab on the AB-strain genetic background, as described earlier by Goldman and using the same construct [12]. The resulting transgene expression exactly recapitulates the original one [12].

Adult zebrafish were raised in a 14 h light∶10 h dark (LD) cycle at 26°C in a multi-tank system (Aquaneering, San Diego, CA, USA). Natural fertilization was scheduled at mid-day, ZT 7 (ZT0 conventionally corresponds to lights-on time) and eggs collected within an hour after (i.e., ZT7-ZT8) were used in individual experiments. Embryos were raised at 26°C, in 14∶10 LD (700∶0 lux) or in constant light (LL, 700 lux) and their ages expressed as hours post-fertilization (hpf) or days post fertilization (dpf).

The chorions were removed 1–2 h before treatment, after which embryos were either randomly distributed between treatment groups or the groups were formed from the embryos of the same clutch (imaging and mortality studies). To assess mRNA expression levels, each sample contained 25 embryos or 20 larvae, and three samples per each treatment group were tested in parallel. All treatment and control groups shown together were processed simultaneously. Embryos were frozen in liquid nitrogen 15 min post-cocaine-treatment.

The experimental protocol was approved by the Boston University Medical School Animal Care and Use Committee.

### Treatments

Treatments were administrated to the embryos at 24 or 36 hours post fertilization (hpf), at ZT 7 or ZT 19, respectively. These ages correspond to 21 hpf and 32 hpf, respectively, when embryos are raised at 28.5°C, [25]. Larval zebrafish were also treated at ZT 7 or ZT 19.

Stock solutions of cocaine (10 mM, NIDA) or melatonin (10 mM, Sigma, St. Louis, MO, USA) were prepared in water. The actual levels of melatonin were confirmed by radioimmunoassay (ALPCO, Windham, NH, USA). Luzindole stock solution (10 mM in ethanol, Sigma, St. Louis, MO, USA) was further diluted in water (50 µM). The treatment or control solutions were added directly into the Petri dish containing the embryos or larvae.

### Image analysis of GFP fluorescence in α1-TGFP zebrafish

For documenting the effects of cocaine on brain development, dechorionated α1-TGFP zebrafish embryos of each clutch were distributed between the treatment groups (30–50 embryos per group). Using embryos of the same clutch reduced intra-group variability in fluorescence. Embryos were raised either in LD or LL, dechorionated and an hour later repeatedly treated for 15 min per hour for 5 consecutive hours (starting ZT 5, at 22 hpf), being washed out after each treatment. The embryos were then examined at intervals after treatment for morphological abnormalities and GFP fluorescence intensity using a fluorescence stereomicroscope (MZFLIII, Leica). Images were captured using a digital camera fitted onto the microscope. Exposure parameters were kept constant throughout each experiment. The GFP fluorescence intensity in specific areas of the original images was quantified using image-analysis software (Image-Pro Plus, 6.2; Media Cybernetics, Inc., Silver Springs, MD). Comparisons were made only between embryos of the same clutch that responded to touch and were similarly positioned in the same field of view in the glass-bottom culture Petri dish.

### Mortality rate evaluation

Change in mortality rate was evaluated following treatments with 1.5 and 30 µM cocaine, administered repeatedly for 15 min per hour for 5 consecutive hours (starting ZT 5, at 22 hpf, and washed out after each treatment), with or without melatonin pre-treatment (100 nM for 20 min). Since different clutches can show substantial differences in survival rate, presumably due to differences in egg quality from different zebrafish parents, paired within-clutch comparisons were used. Prior to treatment, the embryos were dechorionated and each clutch was divided into the control and treatment group (50–80 embryos each). In six clutches with intact chorion, the comparisons were conducted between the control and 30 µM cocaine treatment. The mortality rate was evaluated 24-h after the last cocaine administration. Mortality count in the control group was considered 100% and relative change in this parameter was calculated for the treatment group of the same clutch. The data presented as mean (SEM) percent change, based on the results obtained from six individual clutches per each treatment condition.

### Cocaine levels in larval zebrafish

Zebrafish larvae (40–200 per sample) were treated with cocaine for 15 or 30 min as described above. They were then washed out to remove cocaine from their skin. The control animals were exposed to the same cocaine concentration for 3 sec and then washed out in order to control for the residual amount of cocaine left on larval skin following immersion. The larvae were then fast frozen by immersion in liquid nitrogen. The tissue was homogenized in buffer (0.1 M Sodium Acetate and Sodium Fluoride 1%) and stored at −20°C. All the samples were then sent on dry ice to the Center of Human Toxicology (University of Utah) for further cocaine measurements. There, cocaine levels and those of major cocaine metabolites were measured using 0.5 ml of each sample, followed by the addition of 0.5 ml of blank plasma and the deuterated internal standards (cocaine-d3, benzoylecgonine-d3, and ecgonine methyl ester-d3). Fish samples, along with the calibration standards and quality control samples, were analyzed by solid-phase extraction, followed by liquid chromatography-tandem mass spectrometry (LC/MS/MS) using a electrospray ionization and selected-reaction monitoring [37] with modifications [38]. The resulting lower limit of quantification for each analyte was 2 ng/ml. Protein quantification (BCA Protein Assay, Pierce Biotechnology, Rockford IL, USA) was done for further normalization of cocaine levels measured and the levels are expressed per 100 µg protein.

### Total RNA extraction and cDNA synthesis

25 embryos or 20 larvae were pooled as one replicate sample (N = 3–4 per treatment group), and total RNA was extracted from each replicate using the SV total RNA isolation system (Promega, Madison, WI, USA), according to the manufacturer's protocol. The quantity and quality of RNA was determined spectrophotometrically at 260 nm and 260 nm/280 nm. Same amount of RNA from each sample was converted into cDNA using High-Capacity cDNA Archive Kit (Applied Biosystems, Foster City, CA, USA), according to the manufacturer's instruction.

### Real-time RT-PCR reagents and cycling

Real time quantitative PCR was performed using a TaqMan^®^ Universal PCR Master Mix (ABI, Foster City, CA, USA) and an ABI Prism 7300 Real Time PCR System (ABI, Foster City, CA, USA). TaqMan^®^ probes and primers were designed based on previously reported melatonin sequences of zebrafish and obtained from ABI. The following primers were used:

*zMel1a1*, Forward,5′-CTGGTGATTTTCTCCGTCTACAGA-3′, Reverse, 5′-CCGCCACTGCCAAACTC-3′;*zMel1a-2*, Forward, 5′-TTGGTCATTGTGTCAGTCTTCAGAA-3′, Reverse, 5′-GCTATAGCCAAACTCACCACAAAG-3′; *zMel1a-3*, Forward, 5′-TTGGTGATCTTCTCCGTCTACAGA-3′, Reverse, 5′-TCAGCTACGGCCAGACTCA-3′; *zMel1b-1*, Forward, 5′-TCGGTGTTCAGGAATCGTAAACTG-3′, Reverse, 5′-GAAGGCCAGACTGACCACAAA-3′; *zMel1b-2*, Forward, 5′-TCGGTGCTCCGAAATAGAAAACTC-3′, Reverse, 5′-CGAATGCCAGACTTACTACAAATGC-3′; *zMel1c*, Forward, 5′-CCGTCTACAGGAACAAGAAACTGA-3′, Reverse, 5′-GGTCAGCCACAGAAAGACTCA-3′; *zPer3*, Forward, 5′-CGACTGCCATCCTGGAACTT-3′, Reverse, 5′-GATTTGACCTGCGCACATTCA-3′; *zGH*,, Forward, 5′- GACGGGAAAAGATGAAACGCAAAA-3′, Reverse,5′-TCAATGAGGCGGAAAGAGATACG-3′; *zDAT*,, Forward, 5′-GTCTGGAAGATCTGCCCCATATT-3′, Reverse, 5′- CACATACAGCGAGATCAGGATCAC-3′; *zBmal1*, Forward, 5′-CAGAGCTTCGCCACAAACC-3′, Reverse, 5′-CTGTGATCAATGCATGTCCTTTCA-3′; *β-actin*, Forward, 5′-GCTGTTTTCCCCTCCATTGTTG-3′, Reverse, 5′-TTTCTGTCCCATGCCAACCAT-3′. Gene expression was normalized using a zebrafish β-actin expression level. Each reaction (in triplicate) contained 10 µl TaqMan^®^ Universal PCR Master Mix, 1 µl 20×TaqMan^®^ gene Expression Assay Mix (containing both primers and probes) and 9 µl cDNA diluted in RNase-free water.

### Statistical analysis

RT-PCR data from 3–4 independent samples per treatment group or control were analyzed and fold change was calculated using formula, Fold change = 2^−△(△CT)^, where △C_T_ = C_T(target)_-C_T(β-actin)_ and △(△C_T_) = △C_T(stimulated)_-△C_T(control)_. Student's *t*-tests were used to test the null hypothesis that there was no significant difference between treatment group and control group. The results based on the within-clutch comparisons, e.g., for mortality rate change in six clutches, were analyzed using paired t-test. One-way analysis of variance (ANOVA) was used to compare fluorescence intensity or gene expression between different treatment groups (within each clutch). If significant differences were detected, a Tukey's post-hoc comparison was employed (SPSS, Inc.). In all tests, difference was considered significant if *p*<0.05.
